# Genetic and observational evidence: No independent role for cholesterol efflux over static high‐density lipoprotein concentration measures in coronary heart disease risk assessment

**DOI:** 10.1111/joim.13479

**Published:** 2022-03-22

**Authors:** Sanna Kuusisto, Minna K. Karjalainen, Therese Tillin, Antti J. Kangas, Michael V. Holmes, Mika Kähönen, Terho Lehtimäki, Jorma Viikari, Markus Perola, Nishi Chaturvedi, Veikko Salomaa, Olli T. Raitakari, Marjo‐Riitta Järvelin, Johannes Kettunen, Mika Ala‐Korpela

**Affiliations:** ^1^ Computational Medicine Faculty of Medicine University of Oulu Oulu Finland; ^2^ Center for Life Course Health Research Faculty of Medicine University of Oulu Oulu Finland; ^3^ NMR Metabolomics Laboratory School of Pharmacy University of Eastern Finland Kuopio Finland; ^4^ Biocenter Oulu University of Oulu Oulu Finland; ^5^ Northern Finland Birth Cohorts, Arctic Biobank, Infrastructure for Population Studies Faculty of Medicine University of Oulu Oulu Finland; ^6^ MRC Unit for Lifelong Health and Ageing at UCL Institute of Cardiovascular Science University College London London UK; ^7^ Nightingale Health Plc. Helsinki Finland; ^8^ Medical Research Council Population Health Research Unit University of Oxford Oxford UK; ^9^ Department of Clinical Physiology, Tampere University Hospital and Finnish Cardiovascular Research Center Tampere Faculty of Medicine and Health Technology Tampere University Tampere Finland; ^10^ Department of Clinical Chemistry Fimlab Laboratories and Finnish Cardiovascular Research Center Tampere Faculty of Medicine and Health Technology Tampere University Tampere Finland; ^11^ Department of Medicine University of Turku Turku Finland; ^12^ Division of Medicine Turku University Hospital Turku Finland; ^13^ Research Programs Unit Diabetes and Obesity University of Helsinki Helsinki Finland; ^14^ Estonian Genome Center University of Tartu Tartu Estonia; ^15^ Department of Public Health and Welfare Finnish Institute for Health and Welfare Helsinki Finland; ^16^ Centre for Population Health Research University of Turku and Turku University Hospital Turku Finland; ^17^ Research Centre of Applied and Preventive Cardiovascular Medicine University of Turku Turku Finland; ^18^ Department of Clinical Physiology and Nuclear Medicine Turku University Hospital Turku Finland; ^19^ Unit of Primary Health Care Oulu University Hospital (OYS) Oulu Finland; ^20^ Department of Epidemiology and Biostatistics MRC‐PHE Centre for Environment and Health School of Public Health Imperial College London London UK; ^21^ Department of Life Sciences, College of Health and Life Sciences Brunel University London London UK

**Keywords:** cholesterol efflux, coronary heart disease, genome‐wide association study, HDL, observational cohort study, triglycerides

## Abstract

**Background:**

Observational findings for high‐density lipoprotein (HDL)‐mediated cholesterol efflux capacity (HDL‐CEC) and coronary heart disease (CHD) appear inconsistent, and knowledge of the genetic architecture of HDL‐CEC is limited.

**Objectives:**

A large‐scale observational study on the associations of HDL‐CEC and other HDL‐related measures with CHD and the largest genome‐wide association study (GWAS) of HDL‐CEC.

**Participants/methods:**

Six independent cohorts were included with follow‐up data for 14,438 participants to investigate the associations of HDL‐related measures with incident CHD (1,570 events). The GWAS of HDL‐CEC was carried out in 20,372 participants.

**Results:**

HDL‐CEC did not associate with CHD when adjusted for traditional risk factors and HDL cholesterol (HDL‐C). In contradiction, almost all HDL‐related concentration measures associated consistently with CHD after corresponding adjustments. There were no genetic loci associated with HDL‐CEC independent of HDL‐C and triglycerides.

**Conclusion:**

HDL‐CEC is not unequivocally associated with CHD in contrast to HDL‐C, apolipoprotein A‐I, and most of the HDL subclass particle concentrations.

## Introduction

A functional attribute of high‐density lipoprotein (HDL) particles, cholesterol efflux capacity (CEC), associates inversely with incident cardiovascular events in observational studies, independent of HDL cholesterol (HDL‐C) [1, 2]. However, findings appear inconsistent [3, 4], and it is currently unknown whether HDL‐CEC plays a causal role in cardiovascular disease. No direct randomized controlled trials exist, and Mendelian randomization analyses with reliable genetic instruments are yet to be conducted. Nevertheless, apolipoprotein A‐I (apoA‐I) infusion therapies have failed to show any clinical benefit [5, 6, 7], and a recent Mendelian randomization analysis did not support a cardioprotective role for apoA‐I [8]. These studies provide indirect evidence against the causality of HDL‐CEC since the physiological concept of HDL‐mediated cholesterol efflux is based on the rationale that apoA‐I is the key molecular component to promote cholesterol efflux from arterial wall macrophages [9].

To date, one genome‐wide association study (GWAS) is available with results in 5,293 individuals for four different experimental measures of HDL‐CEC, depicting different efflux pathways, indicating the involvement of five well‐known lipid loci [10]. For the most commonly used HDL‐CEC measure in cardiovascular studies, J774 stimulated HDL‐CEC [1, 3, 4, 11], only two loci were detected, and these associations are not independent of HDL‐C and triglycerides [10].

To clarify the role of J774 stimulated HDL‐CEC in cardiovascular disease, we (1) combined three prospective cohorts (*n* = 14,438) to study the association of HDL‐CEC and various other HDL‐related measures with coronary heart disease (CHD; incident events *n* = 1,570) and (2) performed a GWAS of HDL‐CEC in five independent cohorts of 20,372 participants.

## Methods

### Study populations

The cohorts are characterized in Table 1 (see Study populations in the Supplement for details). The studies were approved by the ethics committees of the study sites and written informed consent was obtained from all participants.

**Table 1:** Characteristics of the cohorts

	FINRISK1997 (*n* = 7,603)	DILGOM2007 (*n* = 4,884)	SABRE (*n* = 3,268)	YFS2007 (*n* = 2,160)	NFBC1986 (*n* = 5,604)	NFBC1966 (*n* = 5,692)
Age, years	48 (37–59)	54 (42–64)	52 (46–58)	39 (33–42)	16 (16–16)	31 (31–31)
Female, *n* (%)	3825 (50)	2602 (53)	465 (14.2)	1186 (55)	2801 (51)	2950 (52)
BMI, kg/m^2^	26.1 (23.5–29.1)	26.5 (24.0–30.0)	25.7 (23.7–28.2)	25.3 (22.7–28.4)	20.5 (19.0–22.6)	24.0 (21.9–26.6)
Systolic blood pressure, mmHg	134 (121–149)	135 (122–149)	122 (112–134)	119 (111–130)	115 (107–124)	124 (115–133)
Diastolic blood pressure, mmHg	82 (74–90)	79 (72–87)	78 (71–85)	75 (68–83)	68 (63–73)	77 (70–84)
Total cholesterol, mmol/L	5.25 (4.55–6.03)	4.30 (3.74–4.87)	3.93 (3.37–4.53)	5.16 (4.56–5.92)	4.29 (3.81–4.88)	5.21 (4.53–6.07)
Triglycerides, mmol/L	1.16 (0.84–1.63)	1.02 (0.78–1.37)	0.92 (0.73–1.19)	1.11 (0.81–1.61)	0.86 (0.67–1.12)	1.02 (0.75–1.44)
LDL cholesterol, mmol/L	1.89 (1.49–2.30)	1.57 (1.27–1.91)	1.40 (1.13–1.70)	1.92 (1.58–2.33)	1.45 (1.19–1.75)	1.89 (1.51–2.34)
HDL cholesterol, mmol/L	1.54 (1.30–1.81)	1.44 (1.23–1.69)	1.01 (0.87–1.17)	1.60 (1.34–1.90)	1.43 (1.24–1.63)	1.59 (1.32–1.90)
HDL‐CEC, %	21.83 (20.66–23.44)	20.37 (19.32–21.73)	21.19 (20.12–22.28)	21.85 (20.84–22.88)	20.43 (19.36–21.37)	21.19 (20.16–22.25)

*Notes*: Data are median (25th and 75th percentiles) or percentage, when appropriate. The number of individuals in each characteristic may vary slightly depending on data availability. Individuals with data available for the required variables were included in each analysis.

HDL‐CEC, HDL‐mediated cholesterol efflux, expressed as an estimated percentage of cholesterol effluxed from total cholesterol present in the cells, as described in Kuusisto et al. [11].

Abbreviations: BMI, body mass index; HDL, high‐density lipoprotein; HDL‐CEC, HDL‐mediated cholesterol efflux capacity; LDL, low‐density lipoprotein

### HDL‐CEC, lipoprotein, and lipid analyses

HDL‐CEC, four HDL subclass particle concentrations, standard lipoprotein lipids, and apoA‐I were analyzed by nuclear magnetic resonance (NMR) spectroscopy [11, 12]. This methodology has been widely used in epidemiological and genetic studies over the last 10 years [12]. The HDL‐CEC values correspond to the most commonly used assay to quantify HDL‐CEC, that is, the use of cAMP‐treated J774 macrophages with radiolabeled cholesterol (J774 stimulated HDL‐CEC) [11]. The HDL‐CEC values as well as all the static HDL‐related measures are directly estimated from the NMR spectral data points with a distinct regression model. Each metabolic measure therefore has its own specific and optimized quantification routine. The estimation of HDL‐CEC does not rely directly on any other HDL (or lipoprotein) measure, and there is no direct simple formula between HDL‐CEC and the other HDL measures [11, 12]. The correlations of the (NMR‐based) HDL‐CEC with other HDL and lipoprotein measures are weak—modest at best—and agree with those using the in vitro HDL‐CEC measurements via cAMP‐treated J774 cell assays [11].

### Statistical analyses in the follow‐up studies

**Fig. 1:**
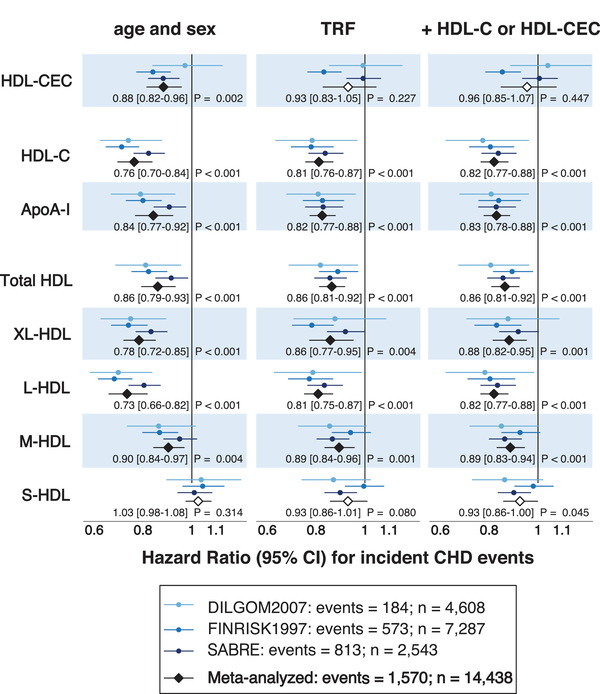
Associations of high‐density lipoprotein (HDL)‐mediated cholesterol efflux capacity (CEC) and various other HDL‐related measures with coronary heart disease (CHD). Data were meta‐analyzed across cohorts by random‐effects meta‐analysis. Hazard ratios are per 1‐standard deviation (SD) higher HDL measure. The HDL subclasses were defined by particle size as follows: very large (XL‐HDL, average particle diameter 14.3 nm), large (L‐HDL, 12.1 nm), medium (M‐HDL, 10.9 nm), and small HDL (S‐HDL, 8.7 nm). Total HDL refers to the sum of all the four HDL subclass particle concentrations. Open and closed black diamonds indicate p ≥ 0.01 and p < 0.01 to denote evidence in favor of an association based on the Bonferroni correction of five independent tests (p = 0.05/5 = 0.01) due to the highly correlated nature of HDL‐related measures.^11^ Traditional risk factors (TRF) included: age, sex, log body mass index, smoking, geographical region (ethnicity in SABRE), diabetes, mean arterial pressure, cardiovascular treatment, and serum concentrations of low‐density lipoprotein cholesterol and log(triglycerides). In the third panel, HDL‐CEC was adjusted for TRF + HDL‐C and other HDL‐related measures for TRF + HDL‐CEC.

Prospective information on CHD events were available for DILGOM2007, FINRISK1997, and SABRE with follow‐up times of 8, 15, and 20 years, respectively. These cohorts had complete data for 14,438 participants with 1,570 incident events (prevalent CHD events, outliers [11], and missing data were removed; see Study populations in the Supplement) and were used to study the associations of HDL‐CEC and other HDL‐related biomarkers with CHD. Data were analyzed by Cox proportional hazard regression models in each cohort and combined via random‐effect meta‐analysis due to obvious heterogeneity in HDL‐CEC associations (Fig. 1).

### Genome‐wide association study

A GWAS of HDL‐CEC was performed in the Finnish cohorts (Table 1) under the additive model, followed by fixed‐effect meta‐analysis (in 20,372 participants) (see Genetic analyses in the Supplement). For comparative purposes, we performed a GWAS of HDL‐C using the same cohorts and participants. In addition to the primary analysis, HDL‐CEC was also analyzed adjusting for HDL‐C and serum triglycerides to directly compare with the findings by Low‐Kam et al. [10]. We also analyzed the associations of the lead single nucleotide polymorphisms (SNPs) of those loci found by Low‐Kam et al. [10] (see Replication of previously described associations in the Supplement).

## Results

The associations of HDL‐CEC and the various HDL‐related concentration measures with incident CHD are illustrated in Fig. 1. There is apparent variation in the associations of HDL‐CEC in the different cohorts. As previously reported [11], in FINRISK1997, the association of HDL‐CEC and CHD is clear with all the various adjustments (including HDL‐C), but this appears not to be the case for the two other population cohorts included in this work. In another Finnish cohort, DILGOM2007, no association is evident between HDL‐CEC and CHD, and the same holds true for the UK cohort, SABRE, when adjusted for traditional risk factors and further with HDL‐C. Thus, in the meta‐analyses, only age‐ and sex‐adjusted HDL‐CEC displayed an inverse association with risk of incident CHD (hazard ratio [HR] 0.88 [95% confidence interval, 0.82–0.96]). This association did not remain after adjustment for traditional risk factors (HR 0.93 [0.83–1.05]) and further adjustment with HDL‐C (HR 0.96 [0.85–1.07]). In contrast, in all the individual cohorts, apoA‐I and all HDL‐related concentration measures associated coherently with incident CHD after corresponding adjustments (HDL‐C was replaced with HDL‐CEC). Thus, in general, the HDL‐CEC results display more heterogeneity between the cohorts than the results for other HDL‐related measures.

**Fig. 2:**
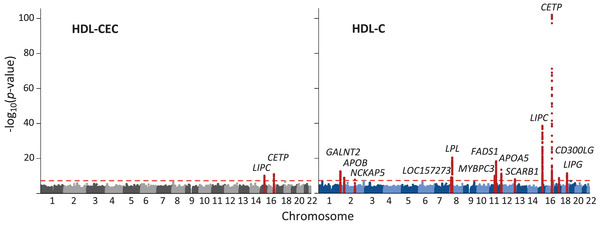
Results of genome‐wide association studies of high‐density lipoprotein (HDL)‐mediated cholesterol efflux capacity (CEC) and HDL cholesterol (HDL‐C). Genome‐wide association studies of HDL‐CEC and HDL‐C were performed in five Finnish cohorts (total n = 20,372). Each dot represents a single single nucleotide polymorphism (SNP); 500‐kb regions flanking the lead SNPs in the associated loci are highlighted. The level of genome‐wide significance (p < 5 × 10^–8^) is indicated by a red dashed line. Altogether two and 13 genome‐wide significant loci were detected for HDL‐CEC and HDL‐C, respectively.

In the GWAS of HDL‐CEC (*n* = 20,372), two loci—hepatic lipase (LIPC; lead single nucleotide polymorphism (SNP) rs261290, *p* = 7 × 10^–11^) and cholesteryl ester transfer protein (CETP; lead SNP rs247616, *p* = 9 × 10^–12^)—associated with HDL‐CEC (Fig. 2, Table S1 in the Supplement). Neither of the loci associated with HDL‐CEC when adjusted for HDL‐C and triglycerides (Figs S1 and S2, Table S1). In contrast to HDL‐CEC, 13 loci associated with HDL‐C in the same set of individuals. The most significant associations colocalized with the HDL‐CEC associations in *LIPC* and *CETP* (Fig. 2, Fig. S3), with the HDL‐CEC increasing alleles being associated with higher concentrations of HDL‐C and the associations being substantially stronger for HDL‐C (rs261290, *p* = 3 × 10^–39^; rs247616, *p* = 3.6 × 10^–102^; Table S1).

Robust causality analyses, neither via univariable nor multivariable Mendelian randomization, were feasible (see Mendelian randomization and assessment of instrument validity in the Supplement).

## Discussion

This is the first study including multiple cohorts with consistent measurements of J774 stimulated HDL‐CEC with the same method. Our large‐scale genetic and observational results—in comparison to multiple HDL‐related concentration measures—suggest that J774 stimulated HDL‐CEC does not unequivocally have an independent role in cardiometabolic risk assessment. These findings fit well with those from other recent studies indicating that HDL‐CEC appears to be a more heterogeneous measure of incident [3, 4] and prevalent [13] cardiovascular outcomes than other HDL‐related biomarkers. Our results also denote more variability between the individual cohorts for the associations of HDL‐CEC and CHD than for those of the other studied HDL‐related measures. While there is a rather contrasting difference in the heterogeneity of the associations for HDL‐CEC and the static HDL measures, the heterogeneity (and its variability) in the observational studies as such is not unexpected. HDL‐CEC estimates functionality that in vivo is most likely a sum of many naturally involved lipoprotein components, that is, it is not inherently HDL specific like the static HDL measures. Thus, the cohort‐specific confounding (e.g., by lifestyle, socioeconomic factors, or baseline health status) affecting the associations of the static HDL measures and HDL‐CEC cannot generally be assumed to be the same. In addition, the potential effects of reverse causation (i.e., when the early stages of the disease process influence the exposure) may vary for different measures [14].

The GWAS for HDL‐CEC performed here is the largest to date with almost four times more participants than in the previous study (20,372 vs. 5,293) [10]. In the primary GWAS, two genetic loci were identified—namely, *LIPC* and *CETP*—but those were abolished when HDL‐CEC was adjusted for circulating HDL‐C and triglyceride concentrations. Previous evidence on the genetic determinants of HDL‐CEC is limited to the study by Low‐Kam et al. [10]. For J774 stimulated HDL‐CEC, they detected two associated loci (*CETP* and *APOE/C1/C2/C4*), but these were not independent of HDL‐C and triglycerides. The *CETP* locus was replicated in our GWAS analyses, but the association was abolished when adjusted for HDL‐C and triglycerides.

We detected 13 associated loci in the GWAS of HDL‐C—performed in the same individuals as the GWAS of HDL‐CEC—suggesting an adequate sample size to detect strong associations. The *LIPC* and *CETP* loci associated with HDL‐CEC in our primary analysis are highly pleiotropic (Table S3), and these associations were abolished via adjustments for circulating HDL‐C and triglycerides. The genetic pleiotropy of HDL‐CEC is not unexpected, since HDL‐CEC is not a single protein biomarker with a clear genetic coding region, but a measure of HDL function, affected inherently by the complexity of overall lipoprotein metabolism.

This large‐scale multicohort study for HDL‐CEC was made possible due to the recent development of a new cost‐effective NMR‐based method to estimate HDL‐CEC directly from serum samples [11]. The estimated HDL‐CEC values have been shown to correspond to those from in vitro experiments, but it cannot be ruled out that the methodology could partly contribute to the heterogeneity between diverse study populations. The results should thus be interpreted with care. It should also be noted that this method is a proxy for radiolabeled cholesterol efflux assay performed in cAMP‐treated J774 macrophages, and it may not be taken to represent other efflux models. The current GWAS for HDL‐CEC with over 20,000 participants—approximately four times more than in the previous GWAS [10]—did not find genetic associations independent of HDL‐C and triglycerides. However, it cannot be ruled out that an even larger GWAS might distinguish HDL‐CEC specific genetic loci or that they might exist for other HDL‐CEC pathways not represented by J774 stimulated HDL‐CEC [11].

The concept behind the pharmacological targeting for cholesterol efflux is based on the idea that agents that increase HDL‐CEC should promote cholesterol removal from atherosclerotic plaques, thereby decreasing the risk for CHD [15]. However, there is no direct evidence that this hypothesis would hold in humans [16]. On the contrary, apoA‐I infusion therapies have not led to reductions in cardiovascular outcomes, despite increasing HDL‐CEC [7]. Furthermore, recent Mendelian randomization analyses have shown that circulating apoA‐I concentrations are unlikely to be causally related to CHD [8, 17], providing indirect evidence that therapeutic modification of HDL‐CEC may not be beneficial.

The current large‐scale genetic and observational analyses temper enthusiasm for an independent role of HDL‐CEC either in disease prediction or as a causal entity in cardiovascular disease. However, further genome‐wide data to potentially allow for robust genetic instruments for HDL‐CEC and Mendelian randomization analyses against various outcomes would be beneficial alongside large‐scale randomized controlled trials. The results and interpretations here regarding HDL function refer only to cholesterol efflux as defined in cAMP‐treated J774 macrophages, and no extrapolation to other efflux models or other types of HDL functionality should be done.

## Author contributions

Sanna Kuusisto, Minna K. Karjalainen, and Mika Ala‐Korpela conceived the idea and drafted the manuscript. Therese Tillin, Antti J. Kangas, Mika Kähönen, Terho Lehtimäki, Jorma Viikari, Markus Perola, Nishi Chaturvedi, Veikko Salomaa, Olli T. Raitakari, Marjo‐Riitta Järvelin, and Mika Ala‐Korpela contributed epidemiological, genetic, or clinical data. Sanna Kuusisto, Minna K. Karjalainen, Therese Tillin, and Antti J. Kangas performed statistical analyses. Michael V. Holmes and Johannes Kettunen contributed to the genetic analyses and formulation of the study concept. All authors provided feedback on the writing of the manuscript. Mika Ala‐Korpela acts as the guarantor for the work.

## Conflict of interests

Antti J. Kangas is employed by Nightingale Health Plc. (Helsinki, Finland), a company providing metabolic profiling services. Veikko Salomaa has consulted for Sanofi and received a modest honorarium from this company. He also has ongoing research collaboration with Bayer Ltd. (all unrelated to the present study). All other authors report no conflict of interests.

## Funding

This study was funded by the EU, Academy of Finland, Social Insurance Institution of Finland, Juho Vainio Foundation, Paavo Nurmi Foundation, Finnish Foundation for Cardiovascular Research, Finnish Cultural Foundation, Sigrid Juselius Foundation; Emil Aaltonen Foundation, Yrjö Jahnsson Foundation, Novo Nordisk Foundation, and others. The funders had no role in study design or reporting.

## Supporting information


**Figure S1**: Manhattan plot showing the results of genome‐wide association study of HDL‐CEC with adjustment for HDL‐C and triglycerides in the five Finnish population cohorts.
**Figure S2**: Regional association plots of the HDL‐CEC associated loci, *LIPC* and *CETP*.
**Figure S3**: Regional association plots of the *LIPC* and *CETP* loci in the GWAS of HDL‐C.
**Figure S4**: Forest plot showing the causal estimates for coronary artery disease.

**Table S1**: SNPs associated with HDL‐CEC in the Finnish populations.
**Table S2**: Associations of previously reported CEC‐associated SNPs with CEC in the Finnish populations.
**Table S3**: Phenotype associations of the HDL‐CEC associated SNPs.
