# Supplementary material for: Genetic and observational evidence: No independent role for cholesterol efflux over static high‐density lipoprotein concentration measures in coronary heart disease risk assessment
Source: J Intern Med. 2022 Mar 22;292(1):146–53. doi: 10.1111/joim.13479 (PMC9311699; doi:10.1111/joim.13479)
Supplement: Supplementary file 1 — Figure S1: Manhattan plot showing the results of genome‐wide association study of HDL‐CEC with adjustment for HDL‐C and triglycerides in the five Finnish population cohorts. Figure S2: Regional association plots of the HDL‐CEC associated loci, LIPC and CETP. Figure S3: Regional association plots of the LIPC and CETP loci in the GWAS of HDL‐C. Figure S4: Forest plot showing the causal estimates for coronary artery disease. [file JOIM-292-146-s002.pdf]

Supplement

Genetic and observational evidence: No independent role for cholesterol efflux over static HDL concentration measures in CHD risk assessment

Sanna Kuusisto, PhD, Minna K. Karjalainen, PhD, Therese Tillin, PhD, Antti J. Kangas, MSc, Michael V Holmes, MD, PhD, Mika Kähönen, MD, PhD, Terho Lehtimäki, MD, PhD, Jorma Viikari, MD, PhD, Markus Perola, MD, PhD, Nishi Chaturvedi, MD, PhD, Veikko Salomaa, MD, PhD, Olli T. Raitakari, MD, PhD, Marjo-Riitta Järvelin, MD, PhD, Johannes Kettunen, PhD and Mika Ala-Korpela, PhD

Online-Only Text

Study populations
- Ethical approvals
- FINRISK1997
- DILGOM2007
- NFBC1966
- NFBC1986
- YFS2007
- SABRE

Genetic analyses
Mendelian randomization and assessment of instrument strength
Replication of previously described associations
STROBE and STREGA statements

eTables

eTable 1: SNPs associated with HDL-CEC in the Finnish populations.
eTable 2: Associations of previously reported CEC-associated SNPs with CEC in the Finnish populations.
eTable 3: Phenotype associations of the HDL-CEC associated SNPs.

eFigures

eFigure 1: Manhattan plot showing the results of genome-wide association study of HDL-CEC with adjustment for HDL-C and triglycerides in the five Finnish population cohorts.
eFigure 2: Regional association plots of the HDL-CEC associated loci, *LIPC* and *CETP*.
eFigure 3: Regional association plots of the *LIPC* and *CETP* loci in the GWAS of HDL-C.
eFigure 4: Forest plot showing the causal estimates for coronary artery disease.

Online-Only References

Online-Only Text

Study populations

The association of HDL-CEC was investigated with incident coronary heart disease in comparison with other HDL-related measures in FINRISK1997, DILGOM2007 and SABRE. Before these analyses, the outliers for each quantitative measure were removed and defined as values that were over 4 times the interquartile range below the 25th percentile and above the 75th percentile.[1] For HDL-CEC, a total of 180 outliers were identified (1.1 % of 15 755 HDL-CEC measurements). Genetic analyses were conducted in five Finnish cohorts (FINRISK1997, DILGOM2007, NFBC1966, NFBC1986 and YFS2007). In the genetic analyses, eligible individuals with genome-wide genotype data available were included from each cohort.

Ethical approvals

The Ethics Committee of the Faculty of Medicine, University of Oulu has approved the Northern Finland Birth Cohort 1986 (NFBC1986) (17.6.1999) and the Northern Finland Birth Cohort 1966 (NFBC1966) studies (17.6.1996). In addition, the Ethics Committee of the Northern Ostro-bothnia Hospital District has approved the NFBC1966 (94/2011) and NFBC1986 (108/2017). The Cardiovascular Risk in Young Finns Study (YFS) was approved by the following Ethics Committees covering all the 5 participating medical university study sites in Finland: the Ethics Committee of the Hospital District of Southwest Finland (12/2007 §533, 19.12.2006; 8/2007 §330, 28.8.2007; 1/2008 §28, 15.1.2008), the Ethics Committee of the Pirkanmaa Hospital District (ETL-R07100), and the Ethics Committee of the Northern Ostrobothnia Hospital District (84/2001). The FINRISK1997 was approved by the Ethics committee of the National Public Health Institute, Helsinki, Finland (23.01.1997), and the DILGOM2007 study was approved by the Ethics Committee of the Helsinki and Uusimaa Hospital District (229/E0/2006). The SABRE study protocols were approved by the University College London (5.1.1988/PMcK/sp) and by the St. Mary's Hospital Research Ethics Committee (07/H0712/109).

FINRISK1997

Since 1972, the FINRISK surveys are carried out in every five years to monitor the health of the Finnish population among persons aged 25–74 years at recruitment in study areas of Finland.[2] In 1997, a total of 8444 persons were recruited in the survey (FINRISK1997). This data was used in the present study. Serum samples were collected in the semifasting state (median fasting time, 5 h; interquartile range, 4–6 h). From initially stored blood samples (n = 8387), the data with HDL-CEC was available for 7603 (91 %) individuals. To study the association of HDL-CEC and other HDL-related measures with incident CHD events (defined as a major coronary heart disease event; acute or subsequent myocardial infarction, unstable angina) individuals with prevalent events (n = 203) and missing data including outliers (n = 113) were removed leaving complete data of 7287 individuals and 573 events for this analysis. A total of 6643 individuals were included in the genetic analyses; genotyping and data processing details have been described before.[3]

DILGOM2007

DIetary, Lifestyle, and Genetic determinants of Obesity and Metabolic syndrome (DILGOM) Survey was conducted as a sub-study of the FINRISK2007 (see FINRISK1997 about general details in FINRISK surveys). Importantly, FINRISK1997 and DILGOM2007 are independent cohorts. DILGOM study was carried out in 5,024 individuals to investigate the factors leading to obesity and metabolic syndrome.[4] Blood samples were collected at 8-hour fasting state. From initially stored blood samples (n = 4977), the data with HDL-CEC was available for 4884 (98 %) individuals. To study the association of HDL-CEC and other HDL-related measures with incident CHD events (defined as a major coronary heart disease event; acute or subsequent myocardial infarction, unstable angina) individuals with prevalent events (n = 148) and missing data including outliers (n = 128) were removed leaving complete data of 4608 individuals and 184 events for this analysis. A total of 3896 individuals were included in the genetic analyses; genotyping and data processing details have been described before.[3]

NFBC1966

The Northern Finland Birth Cohort of 1966 was started to investigate factors affecting preterm birth and subsequent morbidity in the two northernmost provinces in Finland. NFBC1966 included 12 058 children, representing 96% of births in 1966 in data collection region.[5, 6] Data collection on these children continued. Collection at 31 years comprised clinical examination and 12-h fasting blood

sampling for 6007 persons. This NFBC66 collection was used in the present study, including data with HDL-CEC for 5692 (95 %) individuals. A total of 4671 individuals were included in the genetic analyses; genotyping and data processing details have been described before.[3]

NFBC1986

The Northern Finland Birth Cohort of 1986 was carried out during July 1985-June 1986 and included 9,432 births (9,479 children), representing 99% of all the deliveries in the data collection region.[7] Collection at 16 years in 2001-2002 included clinical examination and serum sampling for 6,621 adolescents, representing 71% of invited participants. This collection was used in the present study including data with HDL-CEC for 5604 (85 %) individuals. A total of 3214 individuals were included in the genetic analyses; genotyping and data processing details have been described before.[3]

YFS2007

The Cardiovascular Risk in Young Finns Study (YFS) was conducted to investigate CHD risk factors in children and adolescents of various ages in different areas of Finland.[8] The baseline study was carried out in 1980 and included 3596 children and adolescents aged 3-18. Thereafter, follow-up surveys have been carried out in every ~3 years. In the present study we used the collection at 2007 including 2204 participants, from which data with HDL-CEC was available for 2160 (98 %) individuals. A total of 1948 individuals were included in the genetic analyses; genotyping and data processing details have been described before.[3]

SABRE

The Southall and Brent Revisited study (SABRE) is a community-based cohort from North and West London including 4,858 individuals at baseline from three ethnic groups: Europeans (48%), Indian Asians (35%) and African Caribbeans (16%). Briefly, participants 40 to 69 years of age at baseline (1988 through 1991) were selected randomly from 5-year age- and sex-stratified primary care physician lists (n = 4,063) and workplaces (n = 795) in the London districts of Southall and Brent and traced at follow-up in 2008-2011. Cohort details have been described previously.[9,10] Stored baseline bloods were available only for participants who attended the Southall study clinics (n = 3694) and HDL-CEC measures were available for 3268 (88%) individuals. To study the association of HDL-CEC and other HDL-related measures with incident CHD events (defined as myocardial infarction, acute coronary syndrome, exercise-test confirmed angina and coronary interventions (coronary artery bypass graft, percutaneous transluminal coronary angioplasty, stenting)) individuals with prevalent events (n = 358) and missing data including outliers (n = 367) were removed leaving complete data of 2543 individuals and 813 events for this analysis.

Genetic analyses

A genome-wide association study (GWAS) of HDL-CEC was performed in the five Finnish cohorts (FINRISK1997, DILGOM2007, NFBC66, NFBC86, YFS2007) under the additive model with SNPtest, v2.5.1, followed by fixed-effects meta-analysis with GWAMA, v2.1. The total sample size in this analysis was 20,372. A GWAS of HDL-C was performed using the same cohorts and participants. Before analyses, both phenotypes were first inverse-rank normalized, then adjusted for age, sex, and the first ten principal components (to account for potential confounding caused by population stratification), and inverse rank-based normal transformation was used to transform the resulting residuals to a normal distribution. In addition, HDL-CEC was also analyzed adjusting for HDL-C and serum triglycerides. SNPs with minor allele count <10 or imputation info score <0.8, as well as SNPs not in Hardy-Weinberg equilibrium (p<0.0001), were excluded. Associations with $p < 5\times10^{-8}$ were considered significant. Phenotype associations (diseases and traits, metabolites) of the HDL-CEC associated SNPs were screened using PhenoScanner, v2.[11]

Mendelian randomization and assessment of instrument validity

We assessed the possibility to use HDL-CEC associated SNPs (*LIPC*-rs247616, *CETP*-rs261290) as instruments in Mendelian randomization (MR) analyses. First, we assessed the instrument validity in a univariable setting. With coronary artery disease as the outcome, we assessed the heterogeneity of the causal effects and performed MR with the inverse variance weighted method using the TwoSampleMR package.[12] For coronary artery disease, we utilized summary statistics from the meta-analysis of the UK

Biobank and CARDIoGRAMplusC4D.[13] The mean F statistic (calculated with the formula described in Bowden and Holmes[14]) was 44.9; however, the causal estimates for coronary artery disease were heterogeneous (Q statistic 35, $p=3x10^{-9}$) and the MR estimate was ambiguous (eFigure 4). Consistently, *LIPC* and *CETP* loci are known to be highly pleiotropic (eTable 3), and therefore these SNPs would not present reliable instruments in a univariable setting. We further assessed the possibility to use the HDL-CEC associated SNPs as instruments in multivariable Mendelian randomization (MVMR) analysis including HDL-C, apolipoprotein B and HDL-CEC as exposures and coronary artery disease as the outcome. To this end, we constructed genetic instruments for HDL-C and apolipoprotein B using the TwoSampleMR package,[12] included the HDL-CEC associated SNPs and harmonized the data taking into account LD, which resulted in altogether 88 SNPs. Summary statistics from large GWA studies[13,15,16] were utilized in this analysis. To assess instrument strength, we generated the conditional F statistics using the MVMR R package.[17] When including HDL-C and apolipoprotein B only, the conditional F-statistics were 150 for HDL-C and 13 for apolipoprotein B, respectively, indicative of strong instruments. However, the conditional F-statistic for the HDL-CEC instrument was 1.6 in the multivariable setting. Since a conditional F statistic <10 gives evidence of a weak instrument, this instrument would result in biased MVMR estimates.

Replication of previously described associations

We were also interested to see whether the genetic associations found by Low-Kam *et al.*[18] can be replicated in the current large-scale data. Therefore, we analyzed the association of the HDL-CEC measure with the lead SNPs of loci reported for J774 stimulated HDL-CEC by Low-Kam *et al.* in the current data (eTable2). Low-Kam *et al.* detected associations in two loci (*CETP*, *APOE/C1/C2/C4*) for J774 stimulated HDL-CEC. However, these associations did not remain after adjustment for HDL-C and triglycerides. Associations of the lead SNP in the *CETP* locus (rs247616) was replicated here, but this association was abolished when adjusted for HDL-C and triglycerides. Association of the *APOE/C1/C2/C4* lead SNP (rs445925) was not replicated.

STROBE and STREGA statements

The studies were conducted and reported following the guidelines of the STROBE (STrengthening the Reporting of OBservational Studies in Epidemiology)[19] and STREGA (STrengthening the REporting of Genetic Association studies)[20] statements.

eFigures

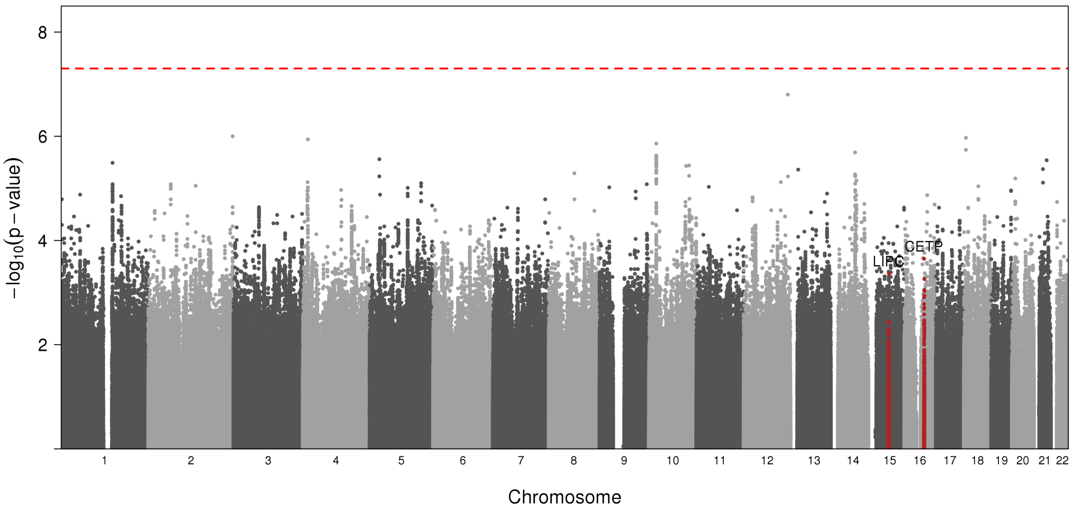

eFigure 1: Manhattan plot showing the results of genome-wide association study of HDL-CEC with adjustment for HDL-C and triglycerides in the five Finnish population cohorts. Each dot represents a single SNP. Chromosomal positions and -log10(*p)* values of each SNP are shown on the X and Y axes, respectively. 500-kb regions flanking the lead SNPs in *LIPC* (rs261290) and *CETP* (rs3764261) in the primary analysis are highlighted.

LIPC

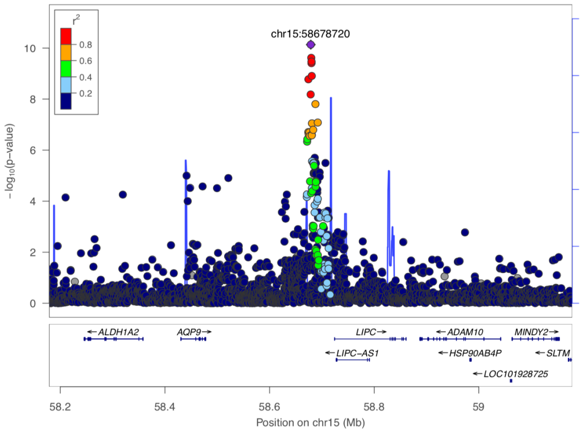

CETP

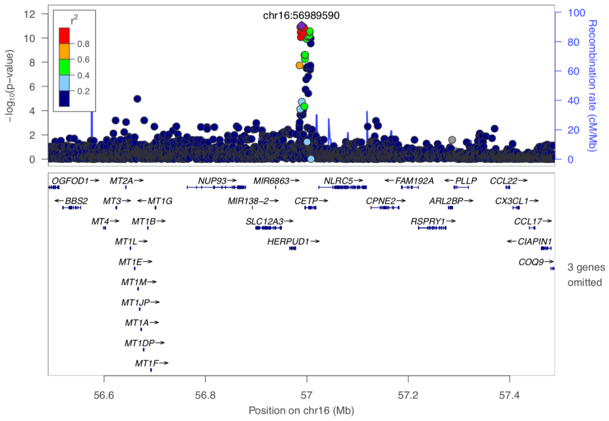

***LIPC*, adjusted for HDL-C and triglycerides**

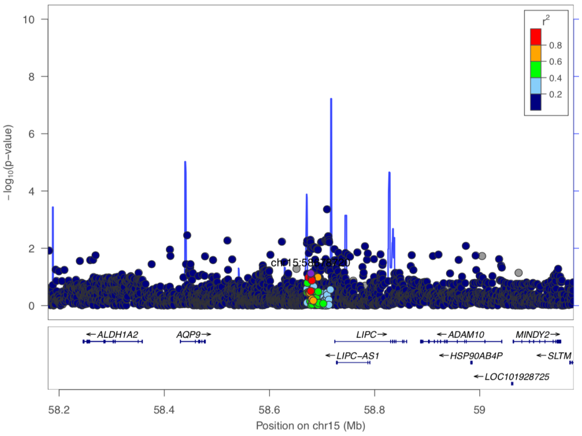

***CETP*, adjusted for HDL-C and triglycerides**

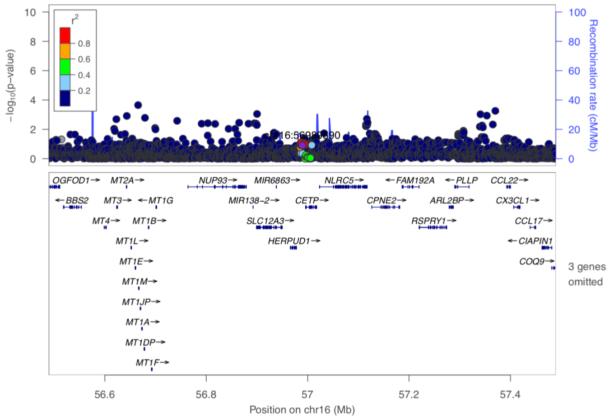

eFigure 2: Regional association plots of the HDL-CEC associated loci, LIPC and CETP. SNPs are highlighted according to linkage disequilibrium (r2) with the lead SNPs (rs261290 and rs3764261, respectively, highlighted in violet).

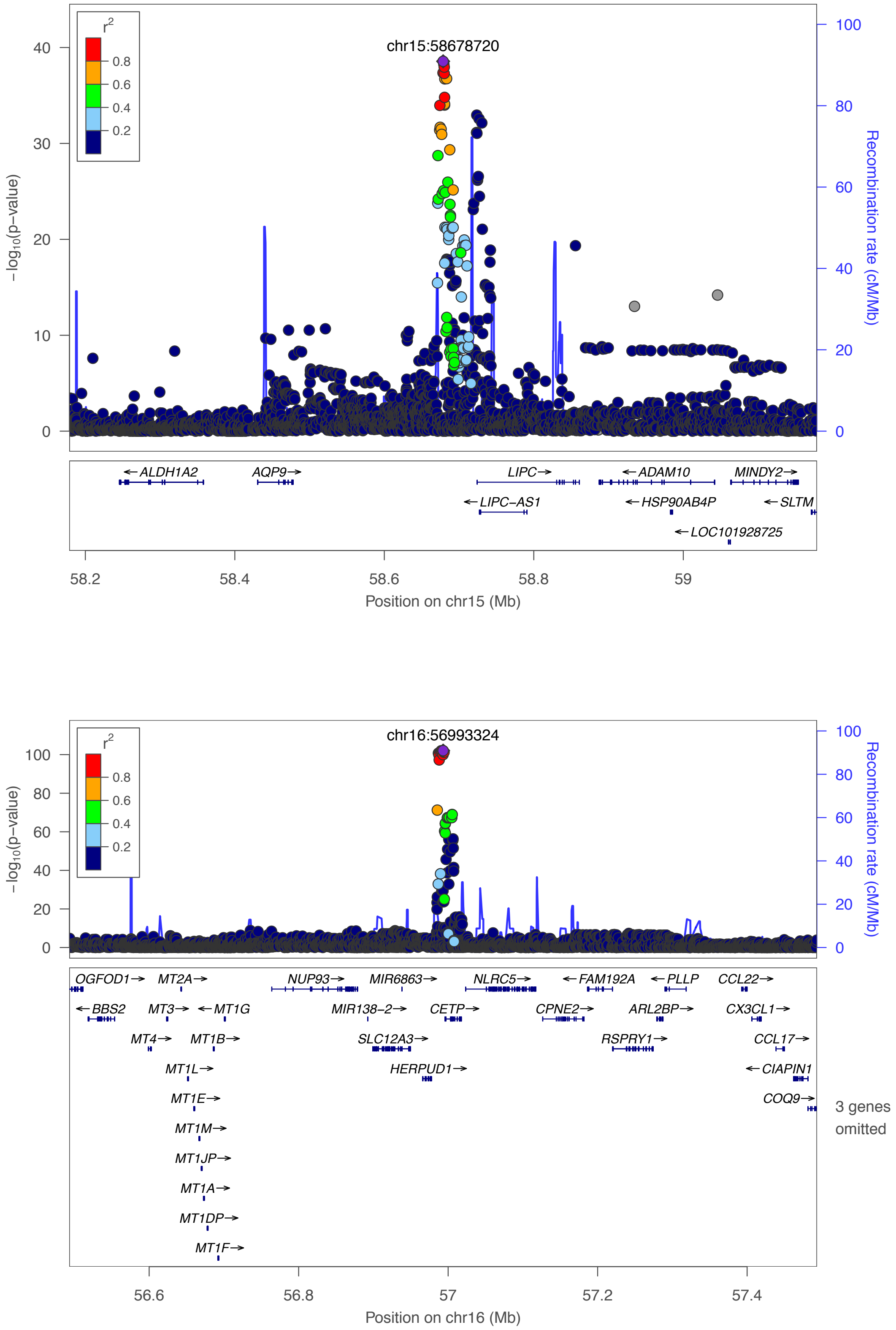

eFigure 3: Regional association plots of the LIPC and CETP loci in the GWAS of HDL-C. SNPs are highlighted according to linkage disequilibrium (r2) with the lead SNP (highlighted in violet) in each associated locus.

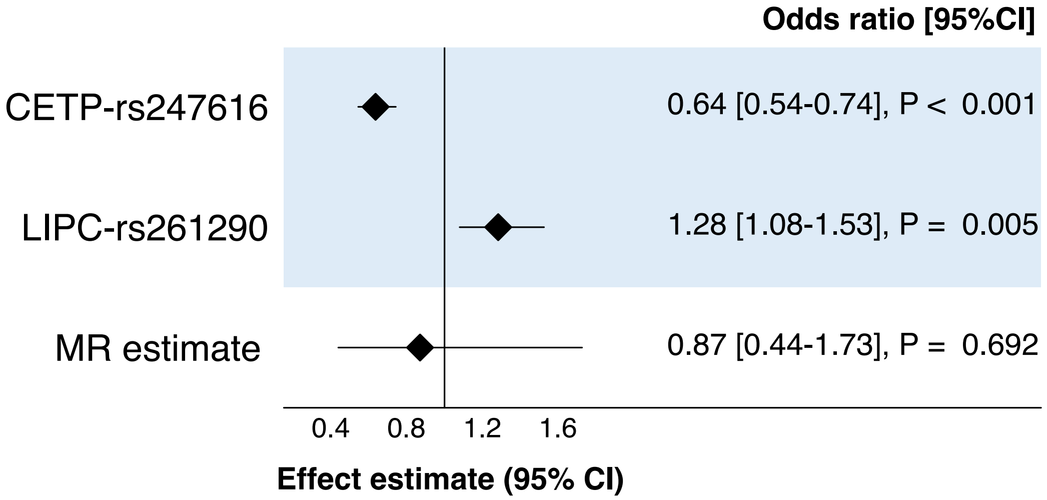

eFigure 4 Forest plot showing the causal estimates for coronary artery disease. Causal estimates were calculated based on associations of *LIPC*-rs261290 and *CETP*-rs247616 (associated with HDL-CEC) with coronary artery disease in meta-analysis of the UK Biobank and CARDIoGRAMplusC4D.[13] Effect estimates correspond to the risk of CAD (OR) per 1-SD increase in HDL-CEC. The associations with coronary artery disease were heterogeneous (Q statistic 35, $p=3 \times 10^{-9}$) and the Mendelian randomization (MR) causal estimate was ambiguous.

Online-Only References

1. Kuusisto S, Holmes MV, Ohukainen P, et al. Direct Estimation of HDL-Mediated Cholesterol Efflux Capacity from Serum. *Clin Chem*. 2019;65(8):1042-1050. doi:10.1373/clinchem.2018.299222

2. Borodulin K, Tolonen H, Jousilahti P, et al. Cohort Profile: The National FINRISK Study. *Int J Epidemiol*. 2018;47(3):696-696i. doi:10.1093/ije/dyx239

3. Karjalainen MK, Holmes MV, Wang Q, et al. Apolipoprotein A-I concentrations and risk of coronary artery disease: A Mendelian randomization study. *Atherosclerosis*. 2020;299:56-63. doi:10.1016/j.atherosclerosis.2020.02.002

4. Konttinen H, Mannisto S, Sarlio-Lahteenkorva S, Silventoinen K, Haukkala A. Emotional eating, depressive symptoms and self-reported food consumption. A population-based study. *Appetite*. 2010;54(3):473-479. doi:10.1016/j.appet.2010.01.014

5. Sabatti C, Service SK, Hartikainen AL, et al. Genome-wide association analysis of metabolic traits in a birth cohort from a founder population. *Nat Genet*. 2009;41(1):35-46. doi:10.1038/ng.271

6. Ollila ME, Kaikkonen K, Jarvelin MR, et al. Self-Reported Polycystic Ovary Syndrome Is Associated With Hypertension: A Northern Finland Birth Cohort 1966 Study. *J Clin Endocrinol Metab*. 2019;104(4):1221-1231. doi:10.1210/jc.2018-00570

7. Järvelin MR, Sovio U, King V, et al. Early life factors and blood pressure at age 31 years in the 1966 northern Finland birth cohort. *Hypertension*. 2004;44(6):838-846. doi:01.HYP.0000148304.33869.ee

8. Raitakari OT, Juonala M, Ronnemaa T, et al. Cohort profile: the cardiovascular risk in Young Finns Study. *Int J Epidemiol*. 2008;37(6):1220-1226. doi:10.1093/ije/dym225

9. Tillin T, Forouhi NG, McKeigue PM, Chaturvedi N, SABRE Study Group. Southall And Brent REvisited: Cohort profile of SABRE, a UK population-based comparison of cardiovascular disease and diabetes in people of European, Indian Asian and African Caribbean origins. *Int J Epidemiol*. 2012;41(1):33-42. doi:10.1093/ije/dyq175

10. Tillin T, Hughes AD, Mayet J, et al. The relationship between metabolic risk factors and incident cardiovascular disease in Europeans, South Asians, and African Caribbeans: SABRE (Southall and Brent Revisited) -- a prospective population-based study. *J Am Coll Cardiol*. 2013;61(17):1777-1786. doi:S0735-1097(13)00789-4

11. Kamat MA, Blackshaw JA, Young R, et al. PhenoScanner V2: an expanded tool for searching human genotype-phenotype associations. *Bioinformatics*. 2019;35(22):4851-4853. doi:10.1093/bioinformatics/btz469

12. Hemani G, Zheng J, Elsworth B, et al. The MR-Base platform supports systematic causal inference across the human phenome. *Elife*. 2018;7:10.7554/eLife.34408. doi:10.7554/eLife.34408

13. van der Harst P, Verweij N. Identification of 64 Novel Genetic Loci Provides an Expanded View on the Genetic Architecture of Coronary Artery Disease. *Circ Res*. 2018;122(3):433-443. doi:10.1161/CIRCRESAHA.117.312086

14. Bowden J, Holmes MV. Meta-analysis and Mendelian randomization: A review. *Res Synth Methods*. 2019;10(4):486-496. doi:10.1002/jrsm.1346

15. Willer CJ, Schmidt EM, Sengupta S, et al. Discovery and refinement of loci associated with lipid levels. *Nat Genet*. 2013;45(11):1274-1283. doi:10.1038/ng.2797

16. Kettunen J, Demirkan A, Würtz P, et al. Genome-wide study for circulating metabolites identifies 62 loci and reveals novel systemic effects of LPA. *Nat Commun*. 2016;7:11122. doi:10.1038/ncomms11122

17. Sanderson E, Davey Smith G, Windmeijer F, Bowden J. An examination of multivariable Mendelian randomization in the single-sample and two-sample summary data settings. *Int J Epidemiol*. 2019;48(3):713-727. doi:10.1093/ije/dyy262

18. Low-Kam C, Rhainds D, Lo KS, et al. Variants at the APOE /C1/C2/C4 Locus Modulate Cholesterol Efflux Capacity Independently of High-Density Lipoprotein Cholesterol. *J Am Heart Assoc*. 2018;7(16):e009545. doi:10.1161/JAHA.118.009545

19. Elm E, Altman DG, Egger M, et al. The Strengthening the Reporting of Observational Studies in Epidemiology (STROBE) statement: guidelines for reporting observational studies. *PLoS Med.* 2007;4(10):e296. doi: 10.1371/journal.pmed.0040296

20. Little J, Higgins JPT, Ioannidis JPA, et al. STrengthening the REporting of Genetic Association Studies (STREGA): An Extension of the STROBE Statement. *PLoS Med.* 2009;6(2):e22. doi: 10.1371/journal.pmed.1000022
